# Pandemic’s Experience Questioning Capitalistic Dominance: Comment on "Ensuring Global Health Equity in a Post-pandemic Economy"

**DOI:** 10.34172/ijhpm.2022.7764

**Published:** 2023-01-04

**Authors:** Alexis Benos

**Affiliations:** Lab of Primary Health Care, General Practice and Health Services Research, Department of Medicine, Aristotle University, Thessaloniki, Greece.

**Keywords:** COVID-19 Pandemic, Public Health, Healthcare, Causes of the Causes, Capitalism

## Abstract

Reflecting on the up-to-date global experience of the coronavirus disease 2019 (COVID-19) pandemic is of crucial importance in order to draw conclusions needed for the design of policies aiming the prevention of new epidemics and the effective protection, preparedness and response of any new emerging. Ongoing environmental destruction, excess mortality by COVID-19 and non-COVID diseases reflecting the dismantlement and commodification of both public health services and healthcare services, deep economic crisis, increasing and deepening social inequalities are the main characteristics raised by the pandemic. The causes of the causes of all these are the dominant rules of the capitalistic system, driven mainly by the unlimited greed for profit on the expenses of the majority of the society. The effectiveness of any proposed correction of this system is discussed and the need for another society responding to the needs of the population is argued.

 Closing its third year, the pandemic’s dramatic impact on the lives and health of the populations globally is nowadays incontestable. Despite the initial approach that the pandemic is a socially neutral disease, continuously growing evidence, during the early months of the pandemic, proved the inequitable context of the coronavirus disease 2019 (COVID-19) pandemic.^1^ The pre-existing social class inequalities that determine both unequal morbidity and mortality distribution, and unequal settings of exposure risks and disease courses, triggered syndemic dynamics with the advent of the pandemic.^2^

 Labonté, in his editorial, is constructively contributing to the international exchange of views trying to answer the crucial, nowadays, question: “Are we eager to return to the “normal” we left behind in early 2020?”^3^ Aiming to ensure global health equity he is thoroughly analysing and discussing different strategies for a different post-COVID-19 economy.

 The recognition, awareness, and concern about social inequalities in health was dramatically risen during the 1990s based on ongoing and growing research evidence.^4^ Consequently, health policy goals included as a priority target the reduction of social inequalities in health, adopting measures mainly addressing the existing socioeconomic gaps.^5^ The next step in this pathway is the emergence and hegemony of the concept of social determinants of health mainly expressed by the homonymous WHO Commission with its 2008 report.^6^ This concept stimulated an academic research movement that produced a wealth of evidence building up the characteristics of concrete factors and showing their association with the unequal distribution of ill-health within the populations. This process generated a shift of the public health research towards the social conditions where people live rather than the still mainstream deterministic biomedical approach. Within this context the fragmented focus and isolation to discreet factors is criticised as a concept that ignores the complexity and multidimensionality of social processes and dynamics.^7^ The social determination of health paradigm was elaborated first by Jaime Breilh introducing the analysis of one holistic social, political, and economic totality.^8^ This approach is tied to a grassroot movement defending collective health and struggling also epistemologically for the decolonisation of science.

 Based on the above-described framework we come back to discuss the question where we want to go after the pandemic experience. It is undisputable that the causes of the causes of the pandemic global tragedy, more clearly than ever, are determined by the hegemony of the market rules, the uncontested dominance of the profit-making in a process of endless economic growth as the main goal and activity of modern society.

 These are the well-known causes of the systemic determination of health: environmental degradation associated with the ongoing climate crisis, expanding multinational profit-making agroindustry, growing socioeconomic inequalities and predictable disease affecting the lower socioeconomic classes which are the great majority of human population. The intensification of social inequalities related to the pandemic, including refugees, asylum seekers, and migrants^9^ are the cynical evidence of this causal chain.

 These are the causes that have driven to the lack of prediction, preparedness and response against an expected emerging pandemic risk, as the non-profit-making public health services and research institutes are suffering after a long standing imposition of austerity policies that produced their structural dismantlement and inertia.

 These are the causes of the unprecedent globally, COVID-19 and non-COVID excess mortality due both to the impotence of the public healthcare services as a result of the commodification process including chronic understaffing and underfunding and the deliberate concealment of the private health sector in order to avoid its infection by COVID-19.^10^

 It is therefore more than clear that the pandemic from its emergence to its inadequate management and dramatic outputs is causally reflecting the systemic characteristics of capitalism.^11^ As part of the same reality, unfortunately, the ongoing year we are experiencing an explosion of the profit-making activities of exploitative capitalism, including a continuing imperialist war that raises the threats of nuclear disaster. And above all we are suffering an antidemocratic shift and authoritarian ruling in global governance, blandly operating for the interests of the few. The management of the procedure of vaccines’ provision dominated by a fundamentalist protectionism of the patents, is a cynical sign of what Benach is calling a “brutal class struggle.”^11^

 Under this perspective it is questionable if the proposals already addressed as solutions to the crisis are applicable. The World Economic Forum’s “great reset to stakeholder capitalism,” the US’s “Build back better,” the European Union’s “green recovery,” and tax and fiscal policy spaces have as an underlying concept the attempt to control the aggressive greed of the capital. As the massive shock, fear and anger expressed globally during the disaster of the first wave of the pandemic did not generate any substantial change of the dominant neoliberal policies, there are no, even theoretical, possibilities for any shift to the values prioritising the needs of the people. This is also cynically proven by the vaccine’s gala. The provisional only, return of the state, as a useful tool for the management of the crisis is showing the unwillingness of the governments to “mitigate capitalism’s inherent inegalitarianism.”^3^ Instead, authoritarian tactics, where applied, are enhancing the way to antidemocratic rule, militarism, and fascism.^12^

 As for the degrowth strategy, if it is not related to systemic changes, it can easily be transformed in another victim blaming and though, disorienting policy that is transferring the blame and the subsequent socioeconomic burden to the poorer countries and to the lower social classes within all countries.

 As Julian Tudor Hart wisely expressed it “…but this will be a redistribution, an intervention to correct a fault natural to our form of society, and therefore incompletely successful and politically unstable, in the absence of more fundamental social change.”^13^

 In conclusion, the COVID-19 pandemic reminded us that it is crucial to readdress health as a commons good and social right, a priority of social justice which can only be granted by democratic governance aiming an equitable and sustainable future.^14^ The occurred tragedy in terms of human lives and social inequalities aggravated by the systemic trend of capitalism to catastrophe are not paving the way to many alternatives. The need to overthrow the ruling capitalist system is realistic, urgent and critical. The crucially important factor is the growing fight, especially by the working class activism, against the luting of the public structures and space and for the prevention of environmental catastrophe and future pandemics. A fight therefore obstructing the return to the previous dominance of profitability and international economic competitiveness.

 The role of the international academic and scientific community is obvious by describing, analysing the situation, revealing the causes of the causes and speaking up for the need of another society built on the aim to cover the needs of the peoples.

## Ethical issues

 Not applicable.

## Competing interests

 Author declares that she has no competing interests.

## Author’s contribution

 AB is the single author of the paper.
